# Development of an Unmanned Aerial Vehicle-Borne Crop-Growth Monitoring System

**DOI:** 10.3390/s17030502

**Published:** 2017-03-03

**Authors:** Jun Ni, Lili Yao, Jingchao Zhang, Weixing Cao, Yan Zhu, Xiuxiang Tai

**Affiliations:** 1 National Engineering and Technology Center for Agriculture/Jiangsu Key Laboratory for Information Agriculture/Collaborative Innovation Center for Modern Crop Production/Jiangsu Collaborative Innovation Center for the Technology and Application of Internet of Things, Nanjing Agriculture University, Nanjing 210095, China; nijun@njau.edu.cn (J.N.); 2015101038@njau.edu.cn (L.Y.); caow@njau.edu.cn (W.C.); 11114416@njau.edu.cn (X.T.); 2 Nanjing Institute of Agricultural Mechanization of National Ministry of Agriculture, Nanjing 210014, China; zhangjc9@163.com

**Keywords:** unmanned aerial vehicle sensor, crop-growth model, computational fluid dynamics, flow field analysis, monitoring system, field experiment

## Abstract

In view of the demand for a low-cost, high-throughput method for the continuous acquisition of crop growth information, this study describes a crop-growth monitoring system which uses an unmanned aerial vehicle (UAV) as an operating platform. The system is capable of real-time online acquisition of various major indexes, e.g., the normalized difference vegetation index (NDVI) of the crop canopy, ratio vegetation index (RVI), leaf nitrogen accumulation (LNA), leaf area index (LAI), and leaf dry weight (LDW). By carrying out three-dimensional numerical simulations based on computational fluid dynamics, spatial distributions were obtained for the UAV down-wash flow fields on the surface of the crop canopy. Based on the flow-field characteristics and geometrical dimensions, a UAV-borne crop-growth sensor was designed. Our field experiments show that the monitoring system has good dynamic stability and measurement accuracy over the range of operating altitudes of the sensor. The linear fitting determination coefficients (R^2^) for the output RVI value with respect to LNA, LAI, and LDW are 0.63, 0.69, and 0.66, respectively, and the Root-mean-square errors (RMSEs) are 1.42, 1.02 and 3.09, respectively. The equivalent figures for the output NDVI value are 0.60, 0.65, and 0.62 (LNA, LAI, and LDW, respectively) and the RMSEs are 1.44, 1.01 and 3.01, respectively.

## 1. Introduction

Real-time, non-destructive, and high-throughput acquisition of crop-growth information is the most important requirement for precision management of crop production. Traditional detection methods which rely on the destructive sampling of plants and indoor physical and chemical analyses, are time-consuming, laborious, and have poor timeliness. In recent years, technologies based on feature recognition using reflection spectra have proven to have several advantages over the traditional methods: non-destructibility, convenient access to information, and good real-time performance. Therefore, this kind of technology has been widely used in research on the mechanisms of monitoring crop growth [1,2,3,4,5,6,7,8,9,10,11].

At present, research institutions around the world have gained access to reflection spectra of crop canopies obtained using various devices (e.g., Cropscan multispectral radiometers [12], ASD FieldSpec 3 hyper-spectrometers [13,14], GreenSeeker sensors [15,16], and CropCircle ACS-470) [17]. The research undertaken has indicated out that there is a good correlation between the reflection spectra of crop canopies and crop nutrients. Hand-held sensors are usually used to statically acquire information about the crop canopy. Although these types of sensors can produce a detailed determination of the spectral characteristics of a crop’s biochemical components, they have several disadvantages including small monitoring range, large labor intensities, and a monitoring regime that is discontinuous. Therefore, these methods cannot provide the high-throughput of information needed for real-time decisions to be made in the production and management of crops spread over large areas in the field. To address this problem, research institutes have started to develop crop-growth monitoring equipment based on vehicle platforms.

The German Yara and Japanese Topcon companies have designed ways to determine the nitrogen content of crops (using their proprietary N-Sensor [18,19] and laser modulated light source sensor CropSpec [20], respectively). In addition, the Trimble Navigation Company based in the United States has also produced the GreenSeeker-RT200 sensor to determine the normalized difference vegetation index (NDVI) of crops [21,22]. Such equipment can continuously gather information on crop growth with a high-throughput and high labor efficiency. However, the vehicle platform causes a certain amount of destruction of crops in operation. Also, operation is not flexible and is easily limited by the size and terrain of the farmland.

The use of unmanned aerial vehicles (UAVs) for such operations promises to have several advantages including high efficiency, good flexibility, convenient operation, and strong adaptability to terrain. Thus, UAVs are becoming more extensively applied to the monitoring of crop growth [23]. By utilizing a miniature hyperspectral infrared thermograph on a UAV, Zarco-Tejada et al. [24] obtained hyperspectral image information on a citrus canopy of large area. The water-stress state of the citrus trees was analyzed offline using remote sensing image processing software including the Environment for Visualizing Images (ENVI). By fixing a color camera onto a UAV, Bendig et al. [25] acquired real-color images of a tree canopy and established a three-dimensional (3D) geometrical model of the trees. Moreover, crop vegetation indices and plant heights could be measured with the use of a ground-based hyper-spectrometer. 

By using UAVs with spiral and fixed wings equipped with a real-color camera and a color-near infrared camera, respectively, Rasmussen et al. [26] obtained information on a crop canopy under different lighting environments. Image processing software was then used to splice and interpret the information obtained so that crop vegetation indices could be obtained. Moreover, it was verified that consumer-grade color cameras could be used to reliably acquire images to allow vegetation indices to be retrieved. A multispectral camera carried on a UAV was used by Caturegli et al. [27] to obtain multispectral images of lawns. By utilizing ENVI software to process the images, information on the vegetation index of the lawns could be extracted to evaluate their nitrogen nutrition status. 

Most of the abovementioned research used a UAV as a platform to carry various types of imaging spectroradiometers to obtain images containing crop information. This information was then corrected offline and spliced using special remote-sensing analysis software in order to interpret the crop-growth information. Due to the complexity of the procedures employed, such an operation needs remote-sensing specialists and is mainly used in scientific research. Furthermore, any possible interpretation of the crop-growth information is delayed and the images cannot be directly used in agricultural production. Besides this, the approach cannot be popularized in agricultural production settings due to the high price of the equipment involved (mainly the various imaging spectrometers).

NDVI and the ratio vegetation index (RVI) are two commonly used indices when inverting crop-growth parameters in the existing UAV-based remote sensing field. Gao et al. [28] carried out experiments using a multi-rotor UAV as the platform from which a crop-growth monitoring system composed of a Canon PowerShot G16 camera and an ADC-Lite multispectral sensor was trialled. In the experiments, remotely sensed images of soybean in its podding and seed-filling stages were obtained. On this basis, by using vegetation indices, including NDVI and RVI, and combining them with LAI data synchronously measured in the field, they constructed univariate and multivariate LAI inversion models using empirical methods. By using an improved camera with an infrared filter borne on an UAV, Ghazal et al. [29] acquired NDVI videos which were then processed to obtain the area of crop growing spots, and relevant agronomic parameters were inverted at the same time. Tian et al. [30] acquired remote sensing images of winter wheat using an UAV-borne ADC air vegetation canopy camera. Based on the spectral characteristics of the images and the changing threshold of NDVI, they proposed a quick classification and extraction method for crops. The results show that, using the method to extract classification information of crops of different types from high-resolution images collected by UAVs presents high accuracy and universality. All this suggests that, while being used for inverting crop agronomic parameters and classifying crop characteristics, NDVI and RVI show high accuracy and potential application value.

In this study, we present a new UAV-borne crop-growth monitoring system based on research achievements of the Nanjing Agricultural University in China relating to crop-growth sensors [31,32,33,34]. The work is aimed at meeting the demands for a high-throughput, continuous, and online real-time method of acquiring crop-growth information. Using a four-rotor UAV (DJI Phantom, SZ DJI Technology Co., Ltd. Shenzhen, China) as the operating platform, we independently design a UAV-borne crop-growth sensor and a matching ground-based data processor to complement the platform. We subsequently used the system to determine, in real-time and online, the major growth indices of a crop canopy including the NDVI, ratio vegetation index (RVI), leaf nitrogen accumulation (LNA), leaf area index (LAI), and leaf dry weight (LDW). This research thus provides a new technological means of acquiring high-throughput growth information for crops covering large areas.

## 2. Design of the UAV-Borne Crop-Growth Monitoring System

### 2.1. Overall Design of the System

**Figure 1:**
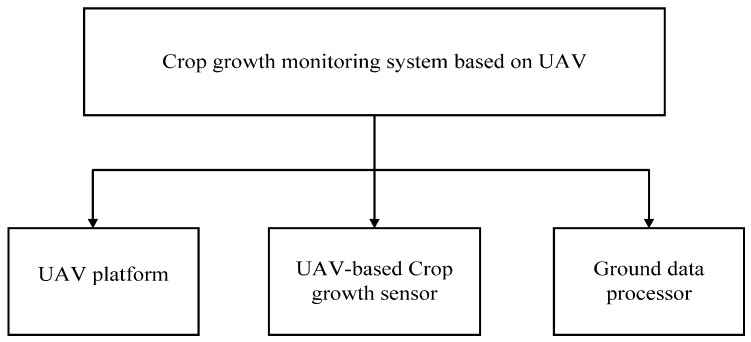
The structure of the UAV-borne crop-growth monitoring system.

The UAV-borne crop-growth monitoring system consists of a UAV platform, a UAV-borne crop-growth sensor, and a ground-based data processor. The UAV-borne crop-growth sensor is fixed to the UAV platform and used to obtain reflection spectra of a crop canopy in real time. The data collected are wirelessly transmitted to the data processor on the ground. The ground-based data processor receives spectral information on the crop canopy which is input into a crop-growth monitoring model. Derived information, including the NDVI, RVI, LNA, LAI, and LDW of the crop canopy, is presented online. The system structure is shown in Figure 1.

### 2.2. Optimization of the UAV Platform

At present, UAVs intended for agricultural use are mainly of the fixed-wing and multi-rotor type [35]. The former drives the aircraft forwards according to the thrust produced by spiral wings or turbine generators. The force of elevation required is generated by the relative movement of the wing and the air. Before flying, the rotors of a UAV need to have a certain initial speed. Common takeoff modes include catapult takeoff and running takeoff. The latter relies on the elevating forces generated by the rotation of multiple spiral wings to balance the weight of the aircraft. This mode does not need the UAV to have an initial speed for takeoff, and is able to realize vertical takeoff. 

**Figure 2:**
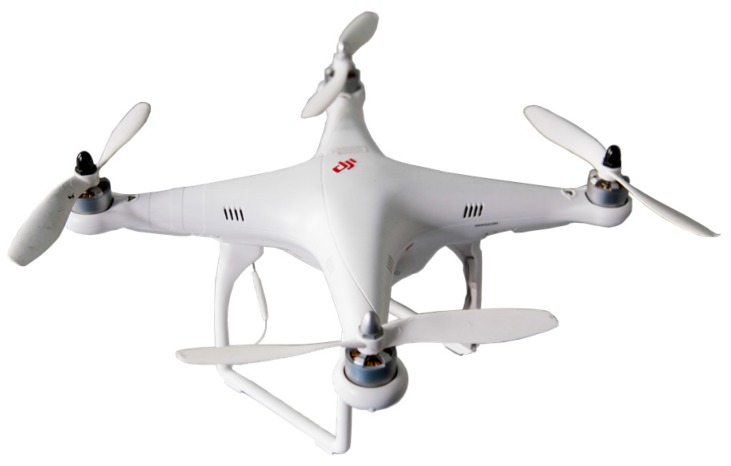
The DJI phantom UAV platform.

Although fixed-wing UAVs are fast and have long cruise durations, the flight speed is hard to adjust according to demand. In addition, they cannot hover and are used merely to carry loads of low weight. In contrast, the flight speed and height of a spiral-wing UAV are adjustable and, in addition, its takeoff mode is simple and places no limits on the takeoff and landing sites [36]. Therefore, spiral-wing UAVs are more suitable for obtaining reflectance spectra of crop canopies in the field. In this study, we used a DJI phantom UAV which is an ideal low-cost platform for use in crop-growth monitoring (Figure 1). The aircraft’s mass, maximum load, flight speed, vertical hovering precision, and horizontal hovering precision are 0.92 kg, 1.2 kg, 10 m/s, 0.8 m, and 2.5 m, respectively. Furthermore, its maximum angular spin velocity, maximum tilt angle, rotor radius, motor speed, and battery life are 1.11 rad/s, 35°, 10.30 cm, 16 r/s, and 18 min, respectively. The DJI phantom UAV is illustrated in Figure 2. 

### 2.3. UAV-Borne Crop-Growth Sensor

The UAV-borne crop-growth sensor consists of a multispectral crop-growth sensor, a sensor support, and a sensor signal-processing circuit. The multispectral crop-growth sensor works on the same measurement principles that ground-based object spectrometers work on. We suppose that the reflection from the crop canopy shows Lambertian reflection characteristics to obtain the bi-directional reflectance of the canopy spectra. Measurement was conducted on sunny days in the absence of heavy cloud cover and strong winds so that the crop canopy remains relatively static. The surface of the crop canopy is close to being a Lambertian reflector and the sensor is placed 1.0–1.2 m directly above the crop canopy in order to capture reflection spectra from it. The sensor support is used for installation of the crop-growth sensor and fixing to the UAV. During low-altitude flight, the rotors of the spiral-wing UAV produce strong airflow fields below the body of the UAV. These may disturb the crop canopy and damage the Lambertian reflectance characteristics. The sensor support ensures that the detection fields of the multispectral sensor relate to the canopy in the absence of airflow disturbance. 

#### 2.3.1. Multispectral Crop-Growth Sensor

The multispectral sensor consists of two kinds of lens (for detection at 720 and 810 nm), which are used to measure the spectral reflectance of the crop canopy. The sensor system utilizes sunlight as the light source which is split using an optical filter. Structurally, there is a solar sensor and a two-band sensor. The former is employed to collect radiation information from the sunlight at 720 nm and 810 nm and to conduct cosine correction. The latter is utilized to collect information on the radiation reflected from the crop canopy at the same wavelengths. 

**Figure 3:**
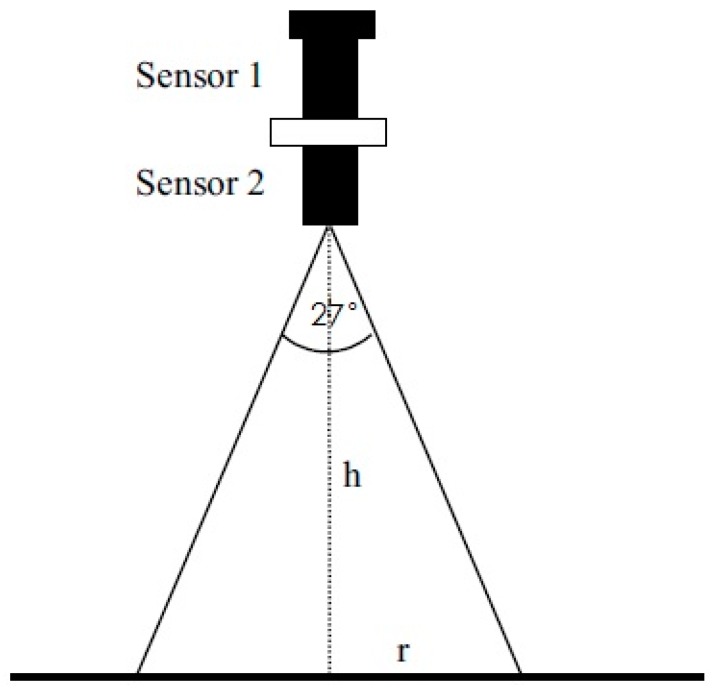
Measurement principles of the multispectral crop-growth sensor. Note: Sensor 1 and sensor 2 represent the solar sensor and two-band sensor, respectively.

The key to designing a multispectral sensor is to determine the correct aperture parameters required for the detection lens. These need to guarantee the sensor system has a high resolution but should also ensure that the signal from the sensor is sufficiently strong. The aperture parameters used in our design are 12.8 mm, 26 mm, and 27°, for the aperture diameter, hole depth, and field of view of the detection lens. The performance parameters correspond to a spectral filter bandwidth of 10 nm and transmittance of 65%–70%. Furthermore, the sensitivity and spectral response of the photoelectric detector selected are 0.55 A/W and 0.011 A/(W/cm^2^), respectively. With this combination of parameters, each detection lens comprises a spectral filter and a photoelectric detector with a simple light path. This guarantees good reliability of the transmitted signal and makes integration and transplantation convenient. The design thus tackles some of the disadvantages of previous crop-growth sensors (complex light paths and heavy use of optical devices). The sensor is packaged within a cylindrical aluminum case, which is highly appropriate for field application. The measurement principles underlying operation of the multispectral sensor are shown in Figure 3.

**Figure 4:**
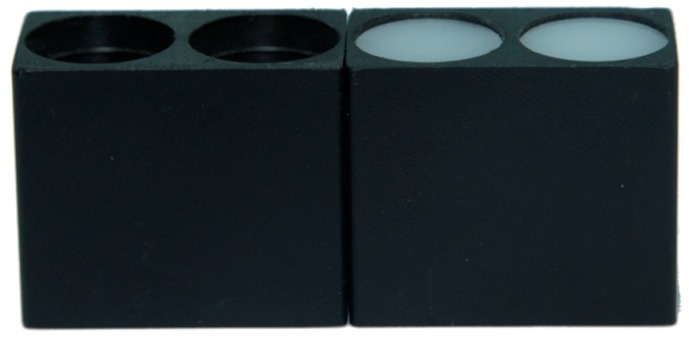
The lightweight structure used for the multispectral crop growth sensor.

As already mentioned, the maximum load of the UAV is 1.2 kg. In order to ensure that the UAV equipped with the multispectral crop-growth sensor is capable of stable flight, the sensor design should be as lightweight as possible. On the premise of retaining the optical structure of the sensor, synthetic fiber (nylon) was adopted in place of the aluminum package holding the sensor to greatly reduce the weight of the sensor (the weight of the improved sensor dropped from 142.7 to 11.34 g, which clearly meets the maximum payload requirements of the DJI Phantom UAV). The structure of the improved sensor is illustrated in Figure 4.

#### 2.3.2. Design of the Sensor Support

The sensor support is used to install the two-band sensor of the multispectral crop-growth sensor to measure the Lambertian reflection intensity of the crop canopy. To ensure measurement accuracy and sensitivity, the working height was set to 1.0–1.2 m above the crop canopy. The canopy also needs to be relatively static. At such low altitudes, the strong down-wash flow fields produced by the spiral wings of the UAV may be expected to affect the sensing process. In particular, the leaves of the canopy can be expected to be displaced towards a common direction under the effects of the airflow field, thus showing Fresnel reflection characteristics and damaging the original Lambertian reflection characteristics of the canopy structure. As a result, the multispectral crop-growth sensor would be unable to correctly obtain the bidirectional reflectance of the canopy spectra. Therefore, the spatial distribution of the down-wash flow field generated by the UAV on the surface of the crop canopy needs to be analyzed. The aim is to determine the optimum length of the sensor support and position of installation of the two-band sensor on the support to ensure that the working field of view of the two-band sensor with respect to the crop canopy is not disturbed by the airflow to a significant extent. 

In recent years, with the rapid development of computer technologies and fluid turbulence models, computational fluid dynamics (CFD) has gradually become a powerful tool for studying the distribution of airflow fields associated with UAVs [37,38]. Here, we use a 3D CFD method of numerical simulation to analyze the distribution of the down-wash flow fields produced by the UAV’s rotors on the surface of the crop canopy. The results are used to propose an optimization scheme for the design of the sensor support.

##### Model Establishment

(1) Physical model of the DJI Phantom UAV

**Figure 5:**
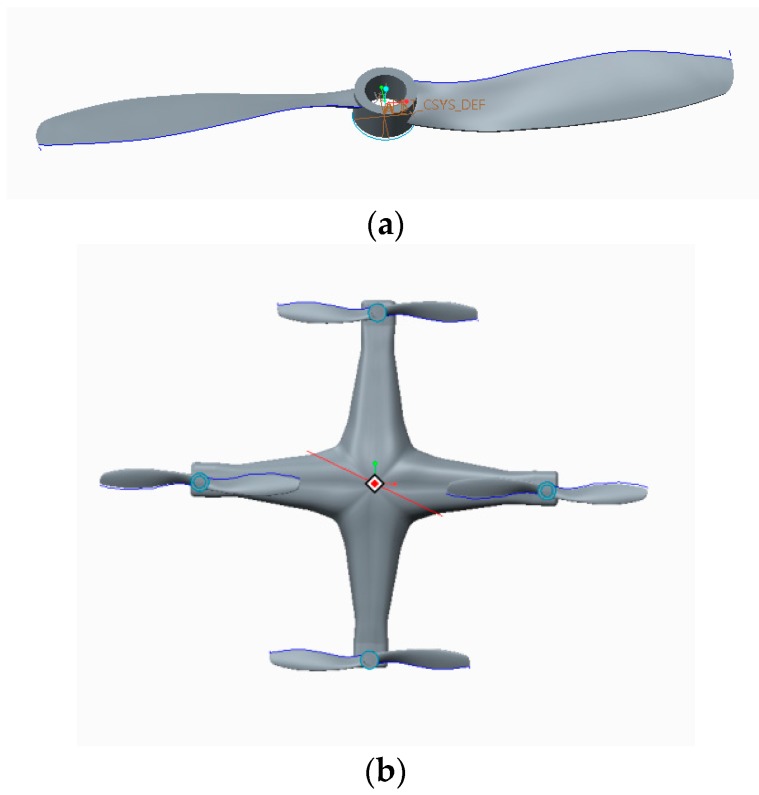
3D models used for the DJI phantom UAV. (**a**) Rotor blade; (**b**) UAV.

The situation is complicated by the irregular nature of the surfaces of the UAV’s rotors, making it difficult to measure linear data. A 3D scanner was therefore used to scan the rotors in order to obtain a uniform point diagram of a rotor blade. By utilizing reverse-engineering software (Imageware, Siemens, Berlin, Germany), the uniform point diagram was then substantialized and the boundaries trimmed to convert it into a blade entity model. Finally, the whole UAV body was modeled (based on measured data) using appropriate modeling software (Creo, Parametric Technology Corporation, MA, USA), and combined with the scanned blade entity, thus obtaining a 3D entity model of the UAV (Figure 5). 

(2) The aerodynamic model

When the UAV hovers, the down-wash airflow exhibits 3D turbulence. The airflow can be described using a series of mass, momentum, and energy conservation equations. We used the standard *k*–*ε* model to solve this problem, resulting in a control equation which can be expressed in the following common form [39,40]:

(1) $$\frac{\partial (\rho \varphi )}{\partial t}+\frac{\partial \left(\rho u\varphi \right)}{\partial x}+\frac{\partial \left(\rho v\varphi \right)}{\partial y}+\frac{\partial \left(\rho w\varphi \right)}{\partial z}=\frac{\partial }{\partial x}\left(\Gamma \frac{\partial \varphi }{\partial x}\right)+\frac{\partial }{\partial y}\left(\Gamma \frac{\partial \varphi }{\partial y}\right)+\frac{\partial }{\partial z}\left(\Gamma \frac{\partial \varphi }{\partial z}\right)+S$$

where *φ*, *Γ*, and *S* represent the generalized variable, diffusion coefficient, and source term, respectively, and *u*, *v*, and *w* indicate the speeds in the *x*, *y*, and *z* directions (m/s), respectively. Furthermore, *ρ* and *t* stand for the density (kg/m^3^) and time (s), respectively. 

##### Numerical Simulations

(1) Grid divisions

The flow fields are stable when the UAV is hovering and the whole of the circumferential flow field tends to be consistent. Therefore, the flow fields of the hovering UAV rotor were simulated as a cylindrical flow field. The calculation domain of this flow field is divided into two parts: an inner, rotating flow field due to the four rotors, and an outer, static flow field which includes the UAV body and airflow fields. The diameter and height of the static field are 1.2 and 1.85 m, respectively, while those of the rotating field are 27.5 cm and 1.8 cm. The UAV rotors are taken to be 1.3 m from the ground. 

The interfaces between the rotating regions in the rotating inner field are axially averaged adopting multiple reference frames, so as to make the values of the flow fields in the circumferential position be identically the same at the same elevation. The interfaces of the rotating regions are rotating walls and the rotating flow field rotates at the rated speed of the UAV rotors in the direction determined by the right-hand rule.

**Figure 6:**
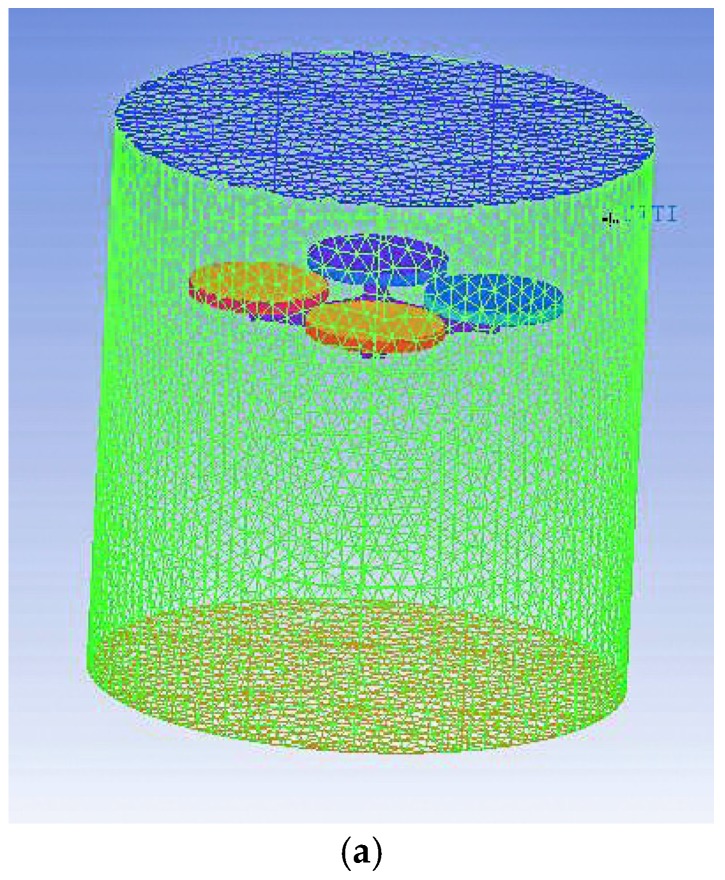

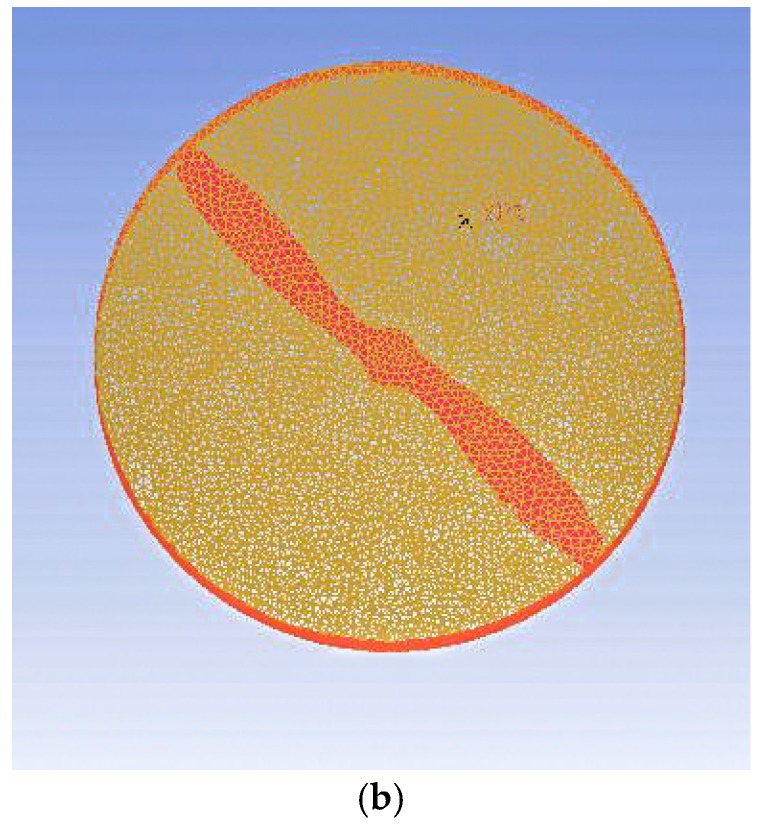
The grid divisions of the flow fields. (**a**) Grid division of the outer flow field; (**b**) Grid division of the inner flow field.

Due to the complex 3D shape of the UAV structure, it is difficult to divide the whole model into structured grids. Thus, we assume that the structure can be reasonably divided into unstructured grids and use the integrated computer engineering and manufacturing code for computational fluid dynamics (ICEM-CFD) to divide the body-fitted grids of the model. Furthermore, the high rotation speeds of the rotor blades leads to a large airflow speed gradient in the inner flow field. However, the outer flow field is only slightly affected. Based on this, dense and sparse grids were generated for the rotor flow field and outer flow field, respectively. In order to improve the accuracy and completeness of data transmission at the interfaces, the grid numbers in the interface regions should be as close as possible. The grid division used is demonstrated in Figure 6.

(2) Boundary conditions

As the UAV is to hover in a flow field corresponding to open space, the outer boundary of the cylinder is set as open. The pressure boundary condition is set to one atmosphere and air at 25° is adopted as the fluid. The rotors are rotating bodies and rotate at a given speed (960 r/min). The interface between the two fields is set as the interface and the reference pressure is taken to be one atmosphere. The roughness of the gas boundary surface is assumed to be zero (i.e., wall without slip). A second-order upwind scheme is utilized for the momentum, turbulent energy, and dissipation equations. To improve the accuracy of the calculations, the residual error is set to have an order of magnitude of 10^−4^.

##### Calculation Results and Analysis

**Figure 7:**
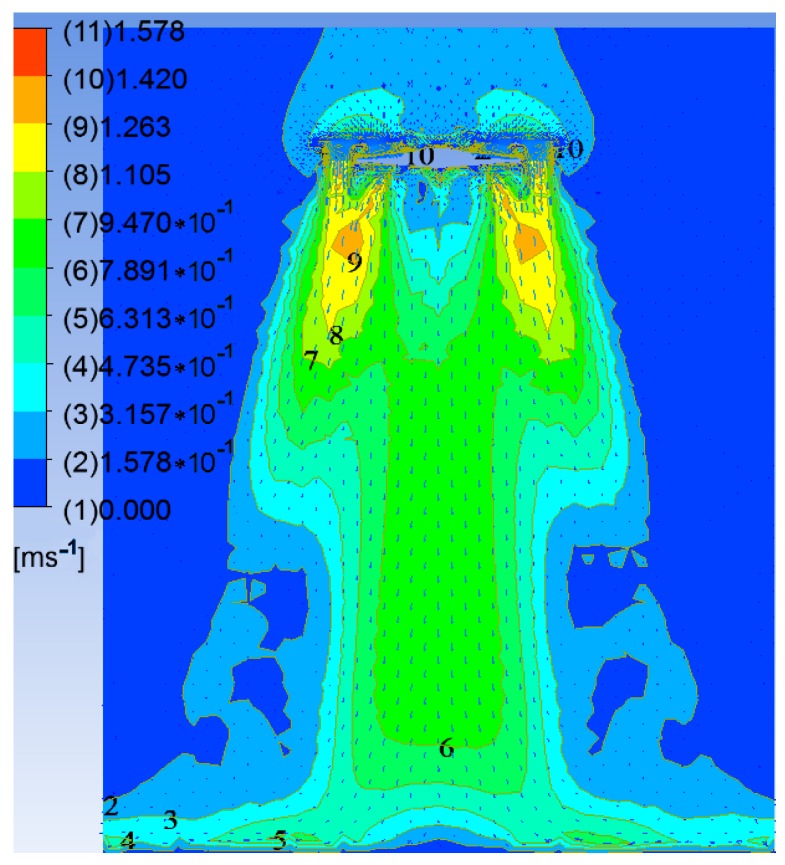
Velocity vector distribution for the down-wash flow fields on the *Z*-*Y* section.

Using the parameters set above, CFX software was used for the numerical calculation and the post-processing modules of the CFX package were utilized for display purposes. Figure 7 shows the distribution of the velocity vectors on the axial section of the down-wash flow fields when the UAV rotors are 1.3 m above the canopy. 

**Figure 8:**
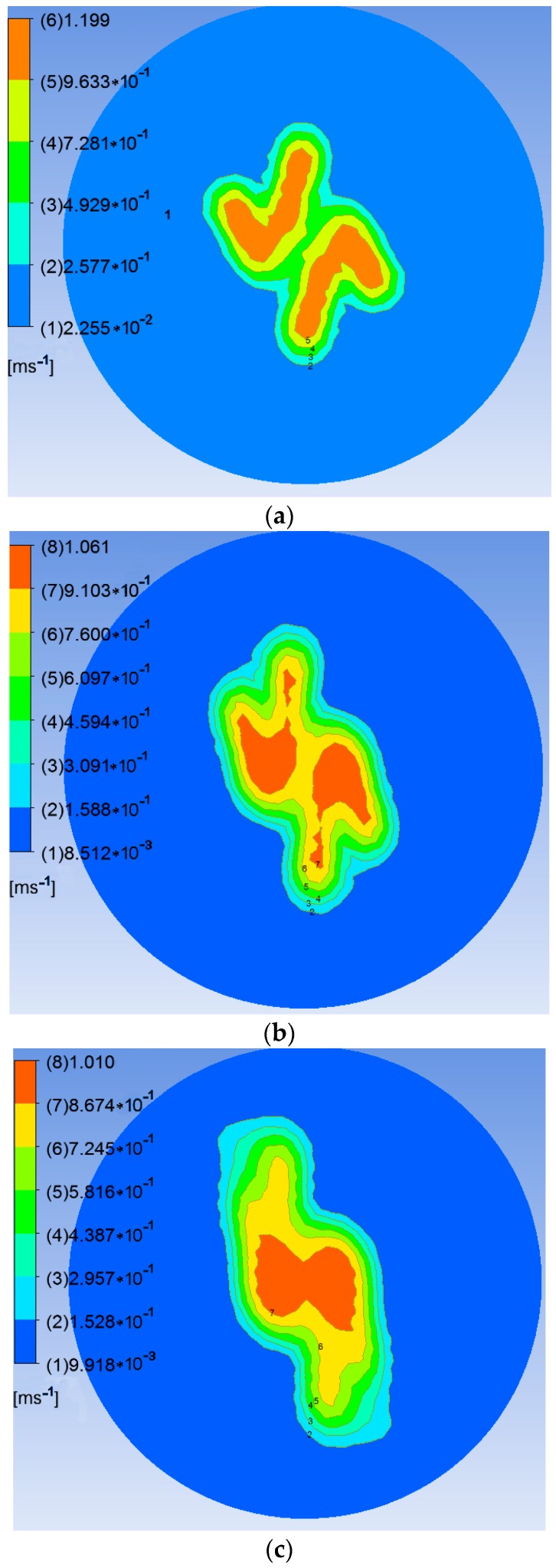

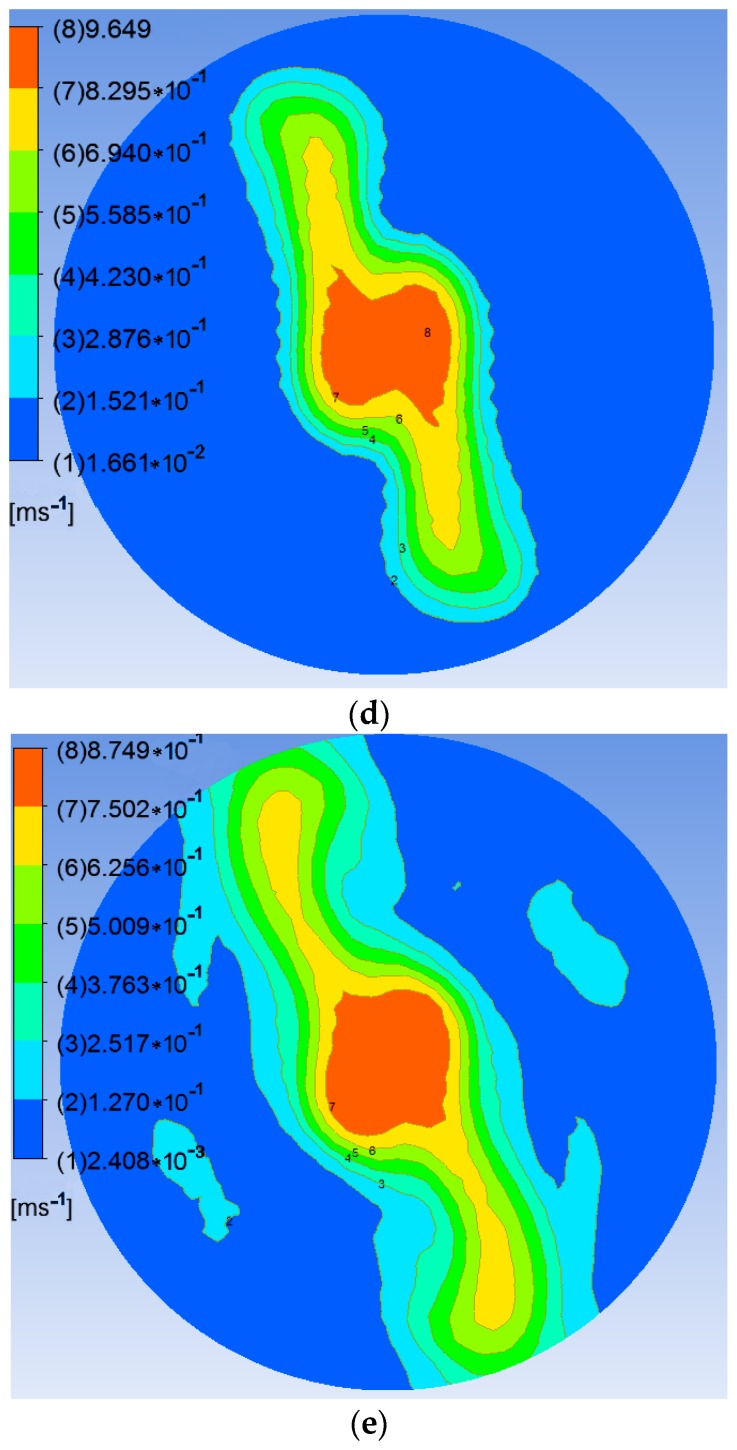
Air velocities in given horizontal planes below the rotors. (**a**) 0.4 m; (**b**) 0.6 m; (**c**) 0.8 m; (**d**) 1.0 m; (**e**) 1.2 m.

Figure 8 illustrates the airflow velocities in horizontal planes 0.4, 0.6, 0.8, 1.0, and 1.2 m below the rotors. 

It can be seen from Figure 7 that the air above the rotors shrinks and sinks under the rotating action of the high-speed rotors. On the one hand, the air flow is thrown outwards by the high-speed rotors. On the other hand, the flow is squeezed by the rotors and forms high-speed flowing regions adjacent to the rotors. The airflow here has high velocity with many axial components. Moreover, the airflow in the down-wash flow fields is concentrated under the rotors and the airflow velocity below the UAV abdomen is significantly slower. After reaching the crop canopy, the airflow spreads around and its velocity falls.

**Figure 9:**
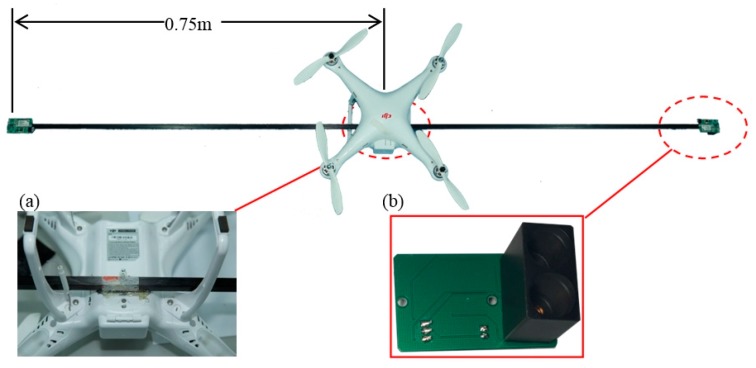
The UAV-borne crop-growth sensor. (**a**) Installation of sensor support; (**b**) Two-band sensor.

As shown in Figure 8, the flow fields in horizontal planes with different elevations below the rotors show basically consistent forms and movement behavior. Because the high-speed rotors affect the down-wash flow fields, a trail is formed in the circumferential direction whose speed gradually decreases. The velocity fields are symmetrical around the central axis and the greater the distance to the central axis, the smaller are the values and gradients of the velocity. The airflow velocities in horizontal surfaces below the rotors gradually drop with increasing vertical distance from the rotors. The maximum speed is 1.20 m/s in the plane 0.4 m from the rotors. This drops to 0.87 m/s in that 1.2 m from the rotors. Furthermore, the area of action of the airflow gradually increases and the maximum width of the flow field is 0.60 m in the horizontal plane 1.2 m from the rotors. Considering the constraints placed on the balance and stability of the UAV, the sensor support should be installed passing through the geometrical center of the UAV abdomen and the solar sensor and two-band sensor should be installed at the two ends of the support. As the field angle of the multispectral crop-growth sensor is 27°, when the operating altitude of the sensor is 1.2 m, the field radius is 0.29 m. To avoid the down-wash flow fields disturbing the crop canopy (considering the maximum width of the down-wash flow fields, the size of the UAV body, and field radius of the sensor), the designed length of the sensor support is set to 1.5 m, as shown in Figure 9.

#### 2.3.3. Sensor Signal Processing Circuit

**Figure 10:**
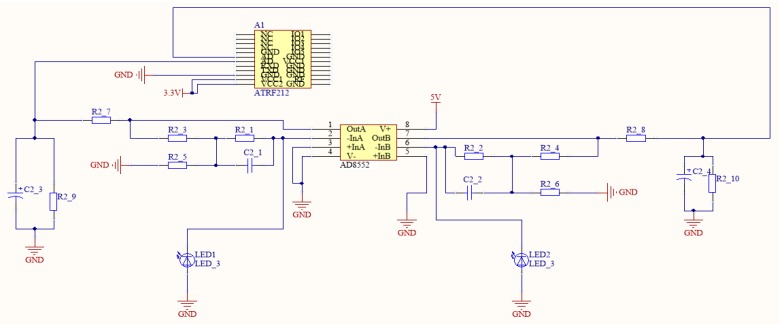
Principles used in the sensor signal processing circuit.

The signal processing circuit carries out photoelectric conversion, amplification, and filtering of the optical information output by the solar sensor and two-band sensor. Then, the characteristic spectral information must be extracted and wirelessly transmitted to the ground-based controller. The circuit therefore includes a photoelectric conversion circuit, an amplifier circuit, a filter and pulse-shaping circuit, and a wireless communication circuit. The radiation of the crop canopy are collected by the two-band sensor and transformed from photonic energy to electrical energy using a photodiode. However, after photoelectric conversion, the electrical signal is very weak. To ensure that the system has high stability and is not likely to be self-excited when the conditioning circuit has a high gain, we designed a T-type amplifier circuit with integral resistance to amplify and filter the electrical signals in this study. The circuit’s principles are displayed in Figure 10.

### 2.4. Ground-Based Data Processor

The ground-based data processor is mainly used to collect and process the signals output by the solar sensor and two-band sensor and to control the two sensors by configuring the wireless communication modules. The processor also calculates certain vegetation indices (RVI and NDVI) and obtains the major growth indices (including LNA, LAI, and LDW) by coupling the crop-growth parameters with the spectral monitoring model. Furthermore, by controlling press keys, the results are displayed on a liquid-crystal display (LCD).

#### 2.4.1. Hardware System

**Figure 11:**
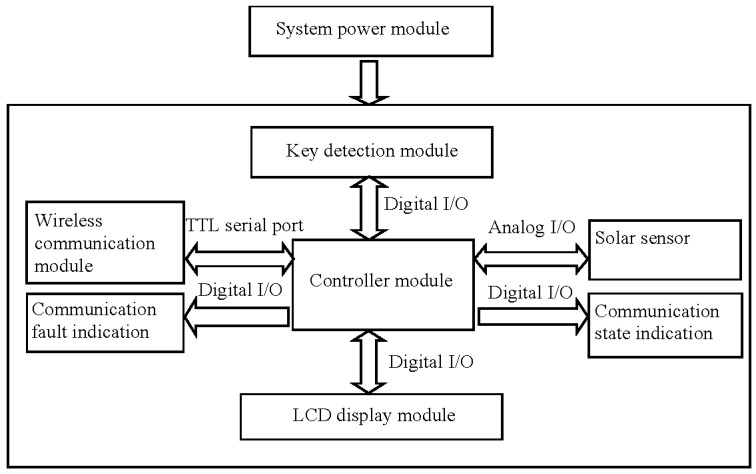
The overall connection structure of the hardware system.

The hardware mainly consists of a controller module, a signal collection module for the solar sensor, a wireless communication module, a key detection module, an LCD display module, and a system power module. An Atmega328P-AU single-chip microcomputer (Atmel, San Jose, CA, USA) was used as the processing core. The controller module receives and processes the data from the solar sensor through a driving analog I/O port. In addition, it drives the digital I/O port to control the key detection module and the LCD display module. Furthermore, by driving the TTL serial ports, the XCBeep wireless communication module can be controlled to receive and send the data collected by the two-band sensor. The communication states and fault test results can be displayed using a light-emitting diode (LED) and configured by the I/O data port of the single-chip microcomputer. The overall connection structure of the hardware system is illustrated in Figure 11.

#### 2.4.2. Software System

The software system is comprised of three modules: a program initialization module, a resource control module for the I/O port, and an application program. The program initialization module was used for power-on self-testing and initialization of the Atmega328P-AU single-chip microcomputer and initialization of the XCBeep wireless communication module (Xiangce Intelligent Technology Co., Ltd., Nanjing, China) and the LCD. The resource control module of the I/O port was utilized for key detection and function switching, LED indication control, and the LCD display. The application program module was employed to collect radiation from the solar and the crop canopy, preprocess the spectral information, calculate the reflectance of the crop canopy, and couple the vegetation indices and crop-growth model. The software system as a whole adopts a modular design, which is convenient for debugging, transplanting, and future upgrades. 

The ground-based controller has function keys in three modes: measurement, calculation, and reset. In the measurement mode, the Atmega328P-AU single-chip microcomputer receives information from the solar sensor through the analog I/O port. The control command “Receiving” is sent to the XCBeep wireless communication module through the serial port to communicate with the two-band sensor. Received spectral information is preprocessed and displayed in real-time. The information obtained by the two-band sensor is transmitted to the ground-based controller (whereupon the LED indicating the communication state flickers at a frequency of 1 kHz). After data transmission, the LED remains lit. When data packets are dropped and lost in transmission, the LED indicating communication faults flickers at a frequency of 1 kHz. At this time, the measurement key needs to be pressed to recollect data from the two-band sensor. 

In the calculation mode, the Atmega328P-AU single-chip microcomputer calculates the major growth indices including the canopy’s spectral reflection, the vegetation’s RVI and NDVI values, which are then coupled with the crop-growth monitoring model to calculate the LNA and LAI of the crops. These indices are displayed on the LCD. In the reset mode, the resources of the controller and the external I/O ports are restored to their initial states.

## 3. Tests and Analysis of Results

### 3.1. Test Design

Systematic field tests were conducted in experimental wheat fields in Sihong County, Suqian City, Jiangsu Province, China from March to May 2016. The test varieties Ningmai 13 and Huaimai 20 were fertilized using five levels of nitrogen application, namely, *N*_0_ (0 kg/hm^2^), *N*_1_ (90 kg/hm^2^), *N*_2_ (180 kg/hm^2^), *N*_3_ (270 kg/hm^2^), and *N*_4_ (360 kg/hm^2^), each of which was repeated three times. Each separate plot covered an area of 42 m^2^ (6 m × 7 m plots). Moreover, 135 kg/hm^2^ of potash fertilizer (K_2_O) was applied so the ratio of nitrogenous to potash fertilizers was 5:5. The basic fertilizers were applied before seeding, while the topdressing fertilizers were fertilized at the jointing stage. In addition, 105 kg/hm^2^ of P_2_O_5_ base fertilizer was used for one time during soil preparation. The other cultivation and management measures undertaken were the same as those commonly employed in high-yield fields.

### 3.2. Test Methods

#### 3.2.1. UAV-Borne Crop-Growth Sensor Measurements at Different Elevations

Before flying the UAV, static tests were carried out. This was done by holding the UAV-borne crop-growth sensor at different elevations to verify the detection performance of the sensor after making the improvement in system weight. The static tests made using the sensor in hand-held mode remove the potential effects of several factors including the shake of the UAV body and rotor wind fields. Tests were carried out at the tillering and jointing stages from 10:00 to 14:00 on sunny days. For each test, the hand-held UAV-borne crop-growth sensor and a commercial ASD spectrometer were simultaneously employed to determine the reflection spectra of the canopy of the wheat. For these measurements, the vertical distance between the two-band sensor and the wheat canopy was either 0.4 or 1.0 m. Three locations in each separate region were measured four times and the average values computed. Then, the NDVI and RVI values determined using the ASD spectrometer at 720 and 810 nm and those output from the UAV-borne crop-growth sensor were recorded.

**Figure 12:**
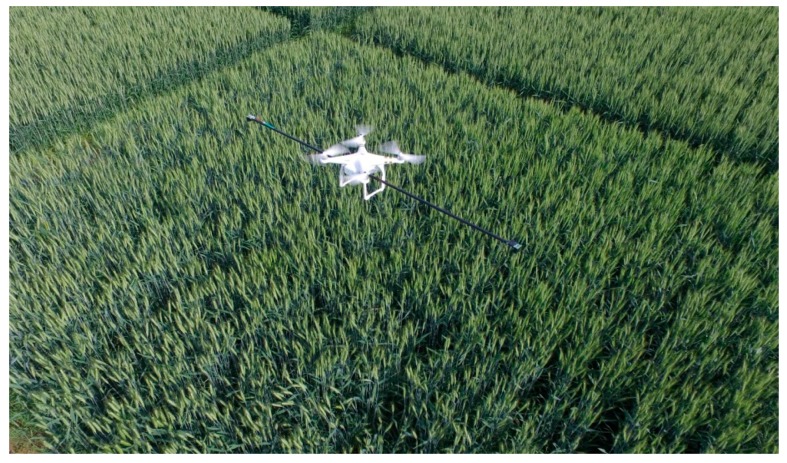
Field tests based on UAV-borne crop-growth monitoring system.

Afterwards, the crop-growth sensor was fixed onto the UAV for the next set of measurements. The initial flight tests made using the UAV/sensor combination were intended to verify the immunity of the designed UAV-borne sensor to the effects of vibration encountered during the flight and disturbance created by the down-wash wind fields from rotors. At the jointing, booting, and heading stages, the reflection spectra of the canopy were dynamically tested at different elevations using the UAV-borne crop-growth sensor from 10:00 to 14:00 on sunny days in the absence of wind. In these tests, the flying height of the UAV was adjusted so that the vertical distance from the two-band sensor to the wheat canopy was 0.4, 0.7, 1.0, and 1.2 m. At each height, the UAV was made to hover by keeping its rotors rotating at the rated speed. In this way, the NDVI and RVI values as output by the UAV-borne crop-growth sensor were recorded at the different elevations used. The field tests are shown in Figure 12.

#### 3.2.2. Performance Tests 

Performance tests were conducted between 10:00 and 14:00 on sunny (windless) days at the tillering, jointing, booting, and heading stages of wheat growth. During the tests, the UAV-borne crop-growth monitoring system was used to measure reflection spectra of the wheat canopies. As a further check, the ASD spectrometer was simultaneously used to detect the reflection spectra of the canopies. The flight height of the UAV was adjusted so that the vertical distance between the two-band sensor and wheat canopy being measured was 1.0 m. Three points in each separate region were measured with four repetitions to permit more representative average values to be calculated. The NDVI and RVI values measured using the ASD spectrometer at 720 and 810 nm and the values output by the UAV-borne crop-growth sensor were recorded. At the same time that the spectral measurements were made, 20 single stems were selected from each region and separated according to their organs in the laboratory. A leaf area meter (model: LAI3000C) was used to measure the leaf area and thereby the LAI of the whole field region could be calculated. Afterwards, the samples were heated to 105 °C for 30 min (as green-killing treatment) and then dried to constant weight at 80 °C. Thus, the LDW could be determined. After smashing the samples, the Kjeldahl nitrogen method was used to determine their LNA values.

### 3.3. Data Analysis

The data from the tests were statistically analyzed by using appropriate software (Excel 2010). The correlation of the model was evaluated by calculating the root mean square errors (RMSEs) and determination coefficients. The stability of the method was assessed through the variances and variation coefficients derived. The necessary formulas required for the calculations are:

(2) $$u=\frac{\sum_{i=1}^{n}x_{i}}{n}$$

(3) $$s=\sqrt{\frac{\sum_{i=1}^{n}{\left(x_{i}-u\right)}^{2}}{n}}$$

(4) $$CV=\frac{s}{u}$$

(5) $$RMSE=\sqrt{\frac{\sum_{i=1}^{n}{d_{i}}^{2}}{n}}$$

In Equations (2)–(5), *x_i_*, *μ*, *s*, *CV*, *d_i_*, and *RMSE* represent the *i*th measured value, mean value, standard deviation, deviation coefficient, difference between the *i*th measured and *i*th true value, and the root mean square error, respectively.

### 3.4. Results and Discussion

#### 3.4.1. Elevation Test Results

**Figure 13:**
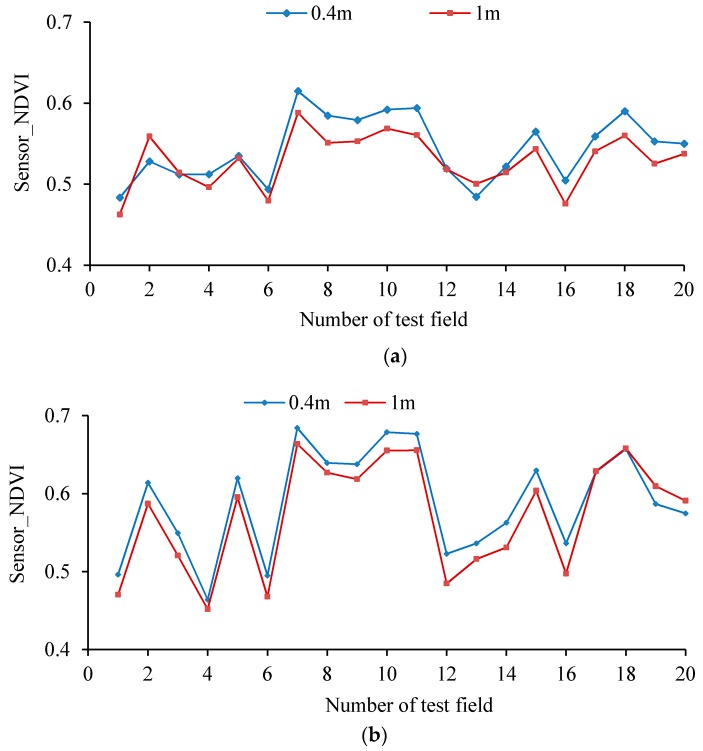

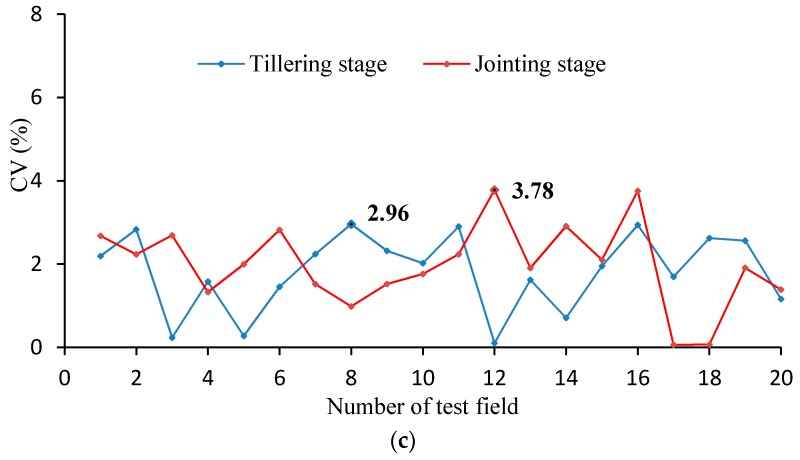
NDVI values measured using the hand-held sensor at different elevations. (**a**) Tillering stage; (**b**) Jointing stage; (**c**) Deviation coefficients of the NDVI values measured.

Figure 13 shows the NDVI values measured when the hand-held crop-growth sensor is 0.4 and 1.0 m from canopy for wheat at its tillering and jointing stages. It can be seen that the variation exhibited by the NDVI curves is consistent at both of the elevations used. By calculating the deviation coefficients, the stability variance of the NDVI values, as measured by the sensor at the two different elevations, is found to be 0.03. The maximum deviation coefficient is 3.78%, so the difference is small. This is a good indication of the high stability of the weight-improved sensor over its intended range of operating altitudes. 

**Figure 14:**
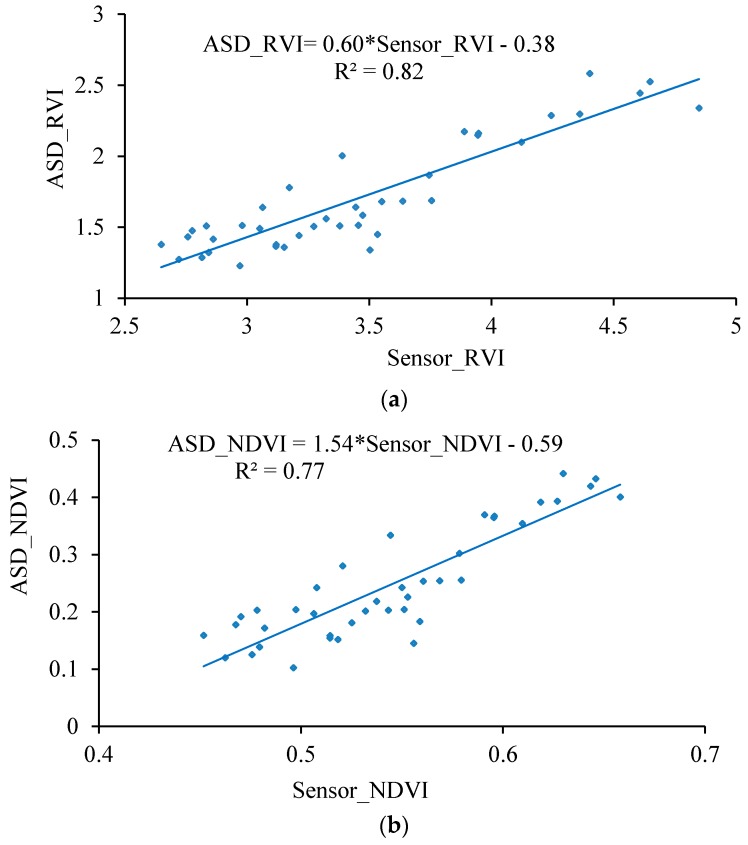
Fitting curves for the hand-held sensor and ASD data. (**a**) NDVI; (**b**) RVI.

The RVI and NDVI values measured by the ASD spectrometer and the UAV-borne crop-growth sensor at an elevation of 1 m (relative to the canopy) were fitted to a one-variable linear function using a least-squares fitting procedure (Figure 14). 

**Figure 15:**
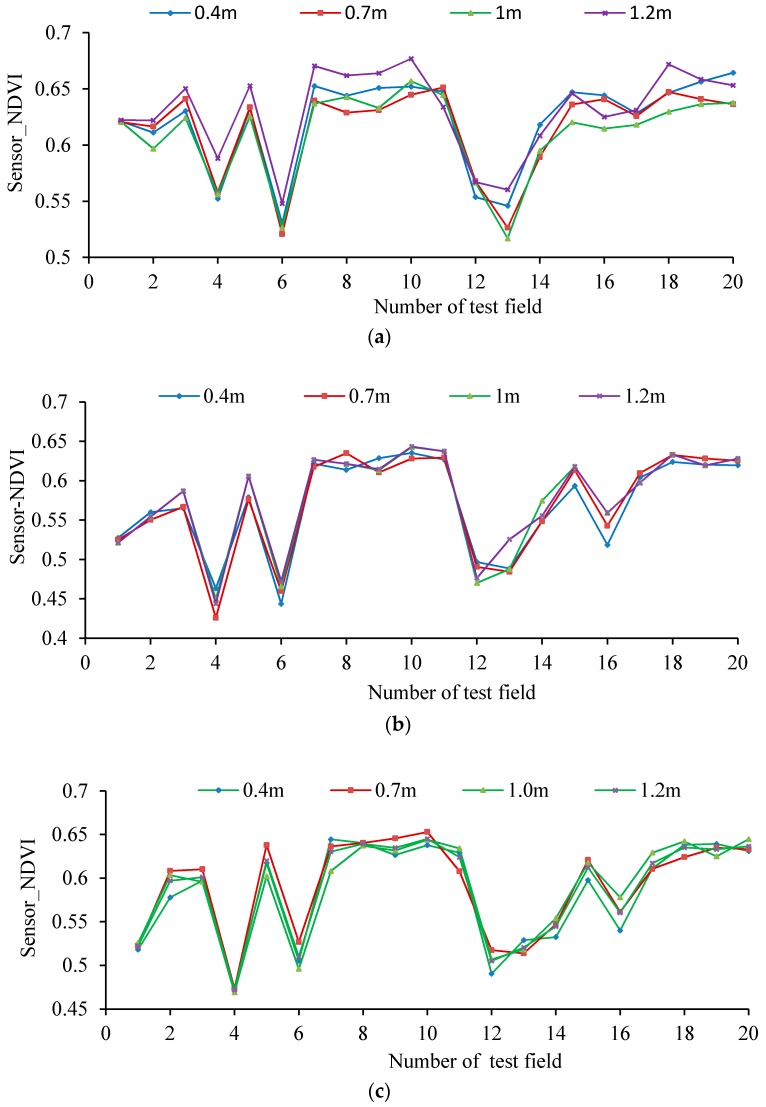

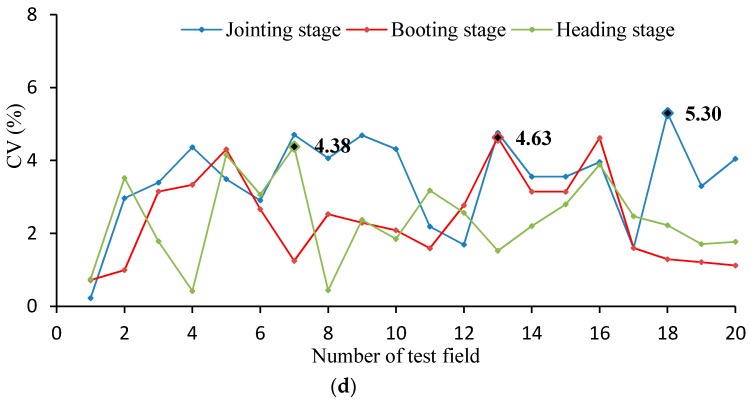
NDVI values measured using the sensor fixed onto the UAV for different elevations. (**a**) Jointing stage; (**b**) Booting stage; (**c**) Heading stage; (**d**) Deviation coefficients of the NDVI values measured.

The figure shows that a good linear relationship exists between the RVI and NDVI values output by the UAV-borne crop-growth sensor and the ASD spectrometer. The coefficients are determined to be 0.82 and 0.77 and the RMSEs are 0.17 and 0.05, respectively. Thus, the measurements made using the lightweight sensor can be seen to have a high level of precision. Figure 15 displays the NDVI values measured when the UAV hovered at 0.4, 0.7, 1.0, and 1.2 m over the canopy (by adjusting to the rated rotation) at the jointing, booting, and heading stages of the wheat. As can be seen from the figure, the changes observed in the NDVI values are consistent at the different measurement elevations employed. The deviation coefficients were then calculated. The variance of stability for the NDVI values measured using the sensor at different elevations is 0.0034 and the maximum deviation coefficient is 5.30%, so the differences are small. This suggests that the sensor installation position is reasonable, and that the effects on the sensor of the UAV vibrations and down-wash flow fields are small. Furthermore, the system has good dynamic stability over the range of operating altitudes of the sensor.

**Figure 16:**
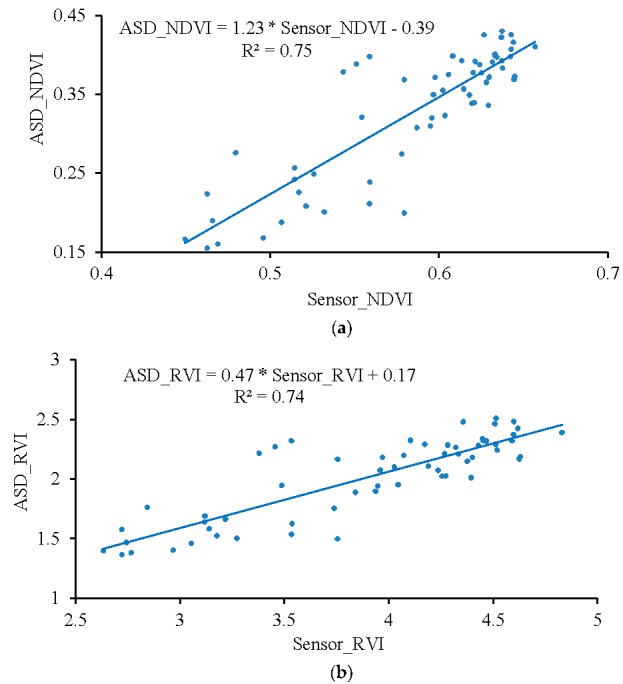
Fitting curves for the UAV-borne sensor and ASD data. (**a**) NDVI; (**b**) RVI.

The RVI and NDVI values measured 1 m from the canopy by the ASD spectrometer and UAV-borne crop-growth sensor were also fitted to a one-variable linear polynomial using least-squares regression (Figure 16). The figure shows there is a good linear relationship between the RVI and NDVI values output by the UAV-borne crop-growth sensor and those from the ASD spectrometer. The coefficients are determined to be 0.74 and 0.75 and the RMSEs are 0.18 and 0.04, respectively. This shows that the sensor support designed according to the numerical simulation of the down-wash flow fields can effectively avoid disturbance from the wind fields. In addition, the UAV-borne crop-growth sensor can be used to make dynamic measurements with high precision. 

#### 3.4.2. Performance Test Results for the UAV-Borne Monitoring System

**Figure 17:**
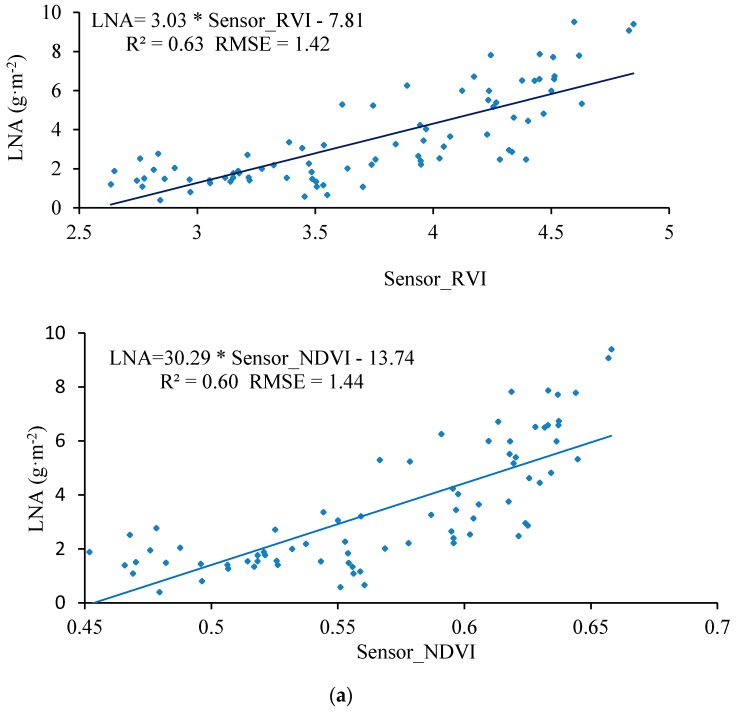

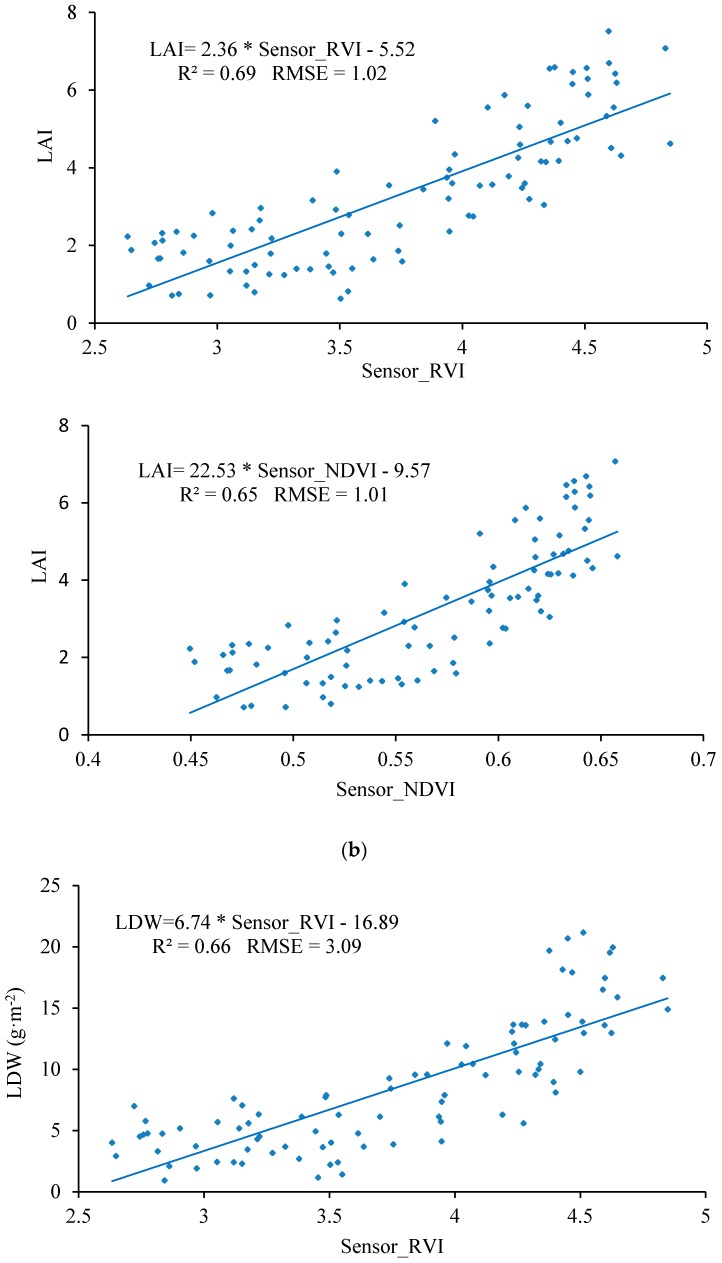

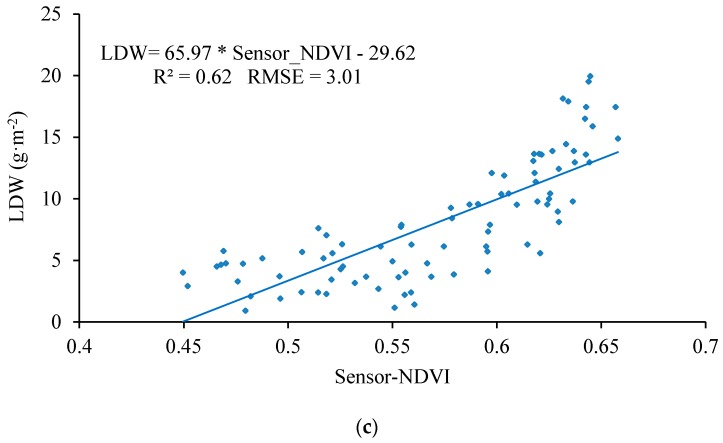
The spectral model for the UAV-borne crop-growth monitoring system. (**a**) LNA-RVI/NDVI fitting curve; (**b**) LAI–RVI/NDVI fitting curve; (**c**) LDW–RVI/NDVI fitting curve.

As shown in Figure 17, the UAV-borne crop-growth monitoring system can accurately reflect the changes in the wheat growth indices (the measured RVI and NDVI values show good linear relationships with LNA, LAI, and LDW). The determination coefficients *R*^2^ of the RVI values with respect to LNA, LAI, and LDW are 0.63, 0.69, and 0.66, and the RMSEs are 1.42, 1.02, and 3.09 respectively. Similarly, the determination coefficients *R*^2^ of the NDVI values with respect to LNA, LAI, and LDW are 0.60, 0.65, and 0.62 and the RMSEs are 1.44, 1.01 and 3.01, respectively. The fitting equations thus established were subsequently stored in the control chips of the ground-based data processor. The system thus calibrated is capable of quickly, non-destructively, and online quantitatively analyzing the growth information subsequently collected on the wheat.

The research developed a UAV-borne crop-growth monitoring system for on-line, and real-time, acquisition of continuous, high-throughput, information about crop growth. Much attention was paid to the design of the matching UAV-borne crop-growth sensor and the crop-growth monitoring system for UAVs. For the former, the key point in the design is to ensure that the working field of view of the downward-looking optical sensor is crop canopies without airflow disturbance. Through CFD simulations, spatial distributions were obtained for the UAV down-wash flow fields on the surface of the crop canopy. Influenced by the high-speed rotation of the rotors, the down-wash flow fields form a trail in the circumferential direction whose speed gradually decreases. When the UAV is 1.2 m from the crop canopy, the maximum velocity is 0.87 m/s on the surface of the crop canopy and the maximum width of the flow field is 0.60 m. Owing to the field angle of the multispectral crop-growth sensor being 27°, the field radius is about 0.29 m when the UAV hovers at 1.2 m above the canopy. In addition, considering the maximum width of the down-wash flow fields, the size of the UAV body, and field radius of the sensor, the sensor support was designed to be 1.5 m long, with which the multispectral crop-growth sensor was integrated with the UAV. It overcomes shortcomings of hand-held multispectral crop-growth sensors such as their small monitoring region, labour-intensity, and discontinuous monitoring: it also improves the test efficiency. As for the UAV-borne crop-growth monitoring system, it needs to be designed to be capable of timeous processing and on-line interpretation of the acquired data. To this end, wireless communication technology is used to transmit information obtained by the UAV-borne crop-growth sensor to the ground-based data processor in real-time. In addition, with the application of a single-chip microcomputer, the information obtained by the sensor and the crop-growth monitoring model is integrated, which overcomes the hysteresis induced by off-line interpretation of existing UAV-borne remote sensing data.

Meanwhile, this new UAV-borne crop-growth monitoring sensor can be operated at two wavelengths, which remains insufficient for the types of vegetation indices required. Therefore the authors plan to develop crop-growth monitoring sensors capable of working at a greater number of wavelengths in future studies, so as to establish more vegetation indices able to predict crop-growth indices and therefore improve prediction accuracy and stability.

## 4. Conclusions

(1) The DJI Phantom quad-rotor UAV can be used as an operating platform to create a matched crop-growth monitoring system. This complete system combines the UAV platform, a UAV-borne crop-growth sensor, and a ground-based data processor. The system can continuously and conveniently obtain the NDVI and RVI values of the crop canopy online (as well as growth indices including LNA, LAI, and LDW) with high throughput and is not limited by the terrain.

(2) Numerical CFD simulations were conducted to investigate the spatial distribution of the down-wash flow fields from the DJI phantom quad-rotor UAV at the surface of the crop canopy. The results show that the airflow is mainly distributed underneath the rotors, and the speed of the airflow below the UAV body is obviously slower. On reaching the crop canopy, the airflow spreads around and its velocity falls. The airflow velocity in horizontal planes below the rotors gradually decreases as the vertical distance from the rotors increases. The maximum velocity is 1.2 m/s at 0.4 m from the rotors and 0.87 m/s at 1.2 m from the rotors. With increasing vertical distance from the rotors, the airflow area gradually increases. The maximum width of the flow field is 0.60 m in the plane 1.2 m from the rotors. On this basis, the length of the sensor support was chosen to be 1.5 m. The solar sensor and two-band sensors were fixed onto the two ends of the support, and this is installed on the UAV so that it passes through the geometrical center of the UAV’s abdomen. This arrangement can effectively avoid the down-wash flow fields below the UAV significantly affecting measurement of the reflection spectra of the crop canopy.

(3) The improved, lightweight UAV-borne crop-growth sensor showed good stability and measurement precision over the range of operating altitudes required of the sensor. When measuring at elevations 0.4 and 1.0 m from the wheat canopy, the stability variance of the NDVI values output by the sensor was determined to be 0.03 and the maximum deviation coefficient was 3.78%. The RVI and NDVI values output by the sensor vary linearly with those obtained by an ASD spectrometer (determination coefficients of 0.82 and 0.77 and RMSEs of 0.17 and 0.05, respectively). Tests of the UAV-borne sensor and UAV show that the designed size and installation position of the sensor support are reasonable and that the effects of in-flight vibration and down-wash are small. Over the operating range of altitudes of the sensor, the monitoring system demonstrated high dynamic stability and measurement precision. When the UAV hovered at 0.4–1.2 m above the canopy (at its rated rotor speed), the stability variance of the NDVI values output by the sensor was determined to be 0.0034 and the maximum deviation coefficient was 5.30%. In addition, the RVI and NDVI values output by the sensor are linearly related to those obtained by the ASD spectrometer (determination coefficients of 0.74 and 0.75, and RMSEs of 0.18 and 0.04, respectively).

(4) The UAV-borne crop-growth sensor performed well when it came to monitoring the growth indices of wheat. The determination coefficients (*R*^2^) of the linear fits between the output RVI values and LNA, LAI, and LDW values were 0.63, 0.69, and 0.66, respectively, and the RMSEs were 1.42, 1.02 and 3.09, respectively. The equivalent figures for the output NDVI values are 0.60, 0.65, and 0.62 (for LNA, LAI, and LDW, respectively), and the RMSEs are 1.44, 1.01 and 3.01, respectively.
